# Polycythemia vera presenting with bilateral papilledema

**DOI:** 10.4103/0301-4738.53067

**Published:** 2009

**Authors:** Suneetha Nithyanandam

**Affiliations:** Department of Ophthalmology, St John's Medical College Hospital, Bangalore, India

Dear Editor,

I read with interest the brief report by Parija *et al*.[1] and appreciate the manner in which the case was diagnosed and treatment instituted leading to good visual recovery in one eye. In this regard, I would like to make the following comments.

The clinical presentation was suggestive of raised intracranial pressure, with the diagnostic workup pointing to cerebral venous thrombosis (CVT). CVT is a relatively common presentation of polycythemia vera.[2] When we reviewed the records of 50 CVT patients treated at our hospital over a period of four years, four cases were secondary to polycythemia vera. Of these four patients three, presented with signs and symptoms similar to the reported patient. In all cases of CVT one should rule out the multiple known causes of CVT including myeloproliferative disorders like polycythtemia vera.[2] Hence, the presentation is not as rare as it has been alluded to in the report.

This report highlights the diagnosis of an uncommon hematological condition which primarily presented to an ophthalmologist. CVT is often under diagnosed even by neurologists. It should be remembered that almost 40% of CVT patients present with signs and symptoms suggestive of isolated intracranial.[3,4] A thorough diagnostic workup including magnetic resonance imaging (MRI) should be done before labeling a case as idiopathic intracranial hypertension (IIH), as the management and outcome of these two conditions vary significantly. With the increasing use of MRI in all cases suspected to be a brain syndrome, CVT has been increasing diagnosed. MRI is now the gold standard in the diagnosis of CVT as rightly done in this case.

Visual loss in CVT maybe due to thrombotic ischemia of any structure of the visual pathway or due to pressure on the optic nerve due to the transmitted raised intracranial pressure (ICP). All cases of CVT with visual loss require visual field analysis and measurement of optic nerve sheath diameter using B-scan ultrasonography (USG). Visual loss in patients with CVT due to transmitted raised ICP (indicated by increased optic nerve sheath diameter on USG) not amenable to medical management is an indication for optic nerve sheath decompression (ONSD). ONSD as a treatment option for the visual loss in the left eye should have been offered to the patient in this case, as it has been shown to be effective even in the presence of optic disc pallor.[4,5] Moreover, ONSD is commonly and more easily done by the medial transconjunctival approach or the lateral orbitotomy approach and not through the orbital roof as mentioned in the report.[1]

## References

[1] Parija S, Mohapatra MM, Pattnaik BK (2008). Polycythemia vera presenting with bilateral papilledema: A rare case report. Indian J Ophthalmol.

[2] Ferro JM, Canhao P, Stam J, Bousser MG, Barinagarrementeria F (2004). ISCVT investigators. Prognosis of cerebral vein and dural sinus thrombosis: Results of the International Study on Cerebral Vein and Dural Sinus Thrombosis (ISCVT) Stroke.

[3] Biousse V, Ameri A, Bousser MG (2000). Isolated intracranial hypertension as the only sign of cerebral venous thrombosis. Neurology.

[4] Lam BL, Schatz NJ, Glaser JS, Bowen BC (1992). Pseudotumour cerebri from cranial venous obstruction. Ophthalmology.

[5] Nithyanandam S, Manayath GJ, Battu RR (2008). Optic nerve sheath decompression for visual loss in intracranial hypertension: Report from a tertiary care center in South India. Indian J Ophthalmol.

